# Supplementary material for: Association between Charlson Comorbidity Index and positive blood cultures at a tertiary-care hospital in Indonesia
Source: PLOS Glob Public Health. 2025 Jun 23;5(6):e0004749. doi: 10.1371/journal.pgph.0004749 (PMC12184916; doi:10.1371/journal.pgph.0004749)
Supplement: Attachment — Submitted filename: Response to Reviewers.docx [file pgph.0004749.s003.docx]

Journal Requirements:

1.  Please provide additional details regarding participant consent. In the ethics statement in the Methods and online submission information, please ensure that you have specified (1) whether consent was informed and (2) what type you obtained (for instance, written or verbal, and if verbal, how it was documented and witnessed). If your study included minors, state whether you obtained consent from parents or guardians. If the need for consent was waived by the ethics committee, please include this information.

If you are reporting a retrospective study of medical records or archived samples, please ensure that you have discussed whether all data were fully anonymized before you accessed them and/or whether the IRB or ethics committee waived the requirement for informed consent. If patients provided informed written consent to have data from their medical records used in research, please include this information.

Thank you. As this is a retrospective study, we have revised the Ethics Approval section to clarify that all data were fully anonymized prior to access and that the requirement for informed consent was waived by the Education and Research Department of Dr. Wahidin Sudirohusodo Hospital.

Additional Editor Comments (if provided):

The authors in this study examined the association between Charlson comorbidity index (CCI) and positive blood cultures among hospital acquired bacteremia cases. The reason for examining this association was to prioritize blood culture testing resources to high yield cases in resource limited settings. To examine their hypothesis, they utilized EMR data between 2015-2018 from a tertiary care hospital in Indonesia. The primary outcome was association between CCI and positive blood culture for any pathogen after adjusting potential confounders and prior antibiotic use as effect modifier. The strength of the study is large database (involving more than 61,000 cases with approximately 3900 patients with blood culture data available) with well thought data analysis. Overall, the manuscript is well written, but I have several methodological clarifications for authors:

1. Lines 118-123- Why were blood culture contaminants not excluded or adjusted for?

Thank you for your comment. In our primary analysis, BC contaminants were categorized as negative because it typically represents organisms introduced during sample collection rather than true bloodstream infections. The BC contaminants were not considered positive because clinical judgment and consideration of patient-specific factors are crucial for accurate interpretation of blood culture results which could not be adequately assessed using available retrospective data. For those reasons, we didn’t exclude or adjust for the BC contaminants.

However, to address this concern, we conducted a secondary analysis focusing only on five priority pathogens (*Staphylococcus aureus, Acinetobacter spp., Klebsiella pneumoniae, Escherichia coli,* and *Pseudomonas aeruginosa*) and the results were consistent with the primary findings.

2. Lines 121 and 136- Authors need to explain by only IV antibiotics were included and not oral antibiotics before blood cultures were taken?

Thank you for your thoughtful comment. We included only intravenous (IV) antibiotics in our analysis because, in our study setting, IV administration is the standard treatment for hospitalized patients with suspected infections. Oral antibiotics are typically prescribed at the time of discharge (following a switch from IV therapy) and are not commonly used before blood cultures are drawn. Additionally, data on oral antibiotic use prior to hospitalization were not available in our dataset. Therefore, we limited our analysis to IV antibiotics to maintain data accuracy and relevance to clinical practice.

3. The other effect modifier would be the specific antibiotic patient was receiving when blood culture was positive. Considering the high antimicrobial resistance among pathogens recovered from positive blood cultures, it is possible that the antibiotic the patients received were selected for the resistant organisms or that the empiric antibiotic therapy was not appropriate. This was discussed in lines 292-293 but I wonder if this could be adjusted in the model.

Thank you for this valuable observation. We agree that the specific antibiotic administered at the time of blood culture collection could influence blood culture positivity, particularly in settings with high antimicrobial resistance. However, due to the complexity of determining the effect of antibiotic related to type, dosage, duration and the type and concentration of pathogens to the outcome, we were unable to adjust for this variable in our multivariable model

4. Lines 327-330: *B. cepacia* is known to cause nosocomial outbreaks and this is an example of potential selection for resistant pathogen despite being on antibiotics. B.cepacia is intrinsically resistant to most beta-lactam antibiotics and therefore the above point I raised is important.

Thank you for highlighting this point. Further work is needed to better understand *B. cepacia* clinical (true pathogens vs. potential contaminant) and epidemiological significance.

5. As authors state there are several issues studying the association of CCI with positive blood culture in an LMIC setting like the authors used- most of patients receive antibiotics prior to blood cultures, bias in selecting patients where blood cultures will be obtained, poor infection control practices, high blood culture contamination rates, nosocomial outbreaks. So, the question is whether this was even an appropriate setting to examine this question. Maybe, we should examine this association in high-income settings where the influence of the factors mentioned above is minimal.

Thank you for this important observation. We acknowledge the challenges inherent in studying the association between comorbidities and blood culture positivity in LMIC settings, including prior antibiotic exposure, selective testing practices, infection control limitations, and higher contamination rates. However, these very factors represent the realities of clinical practice in LMICs and highlight the complexity of diagnostic decision-making in resource-constrained environments. We believe it is essential to explore such associations in LMIC contexts, as findings from high-income settings may not be generalizable. While our results did not demonstrate a clear association, they underscore the need for context-specific strategies to improve diagnostic stewardship and blood culture utilization in LMICs.

Reviewers' comments:

Reviewer's Responses to Questions

**Comments to the Author**

1. Does this manuscript meet PLOS Global Public Health’s [publication criteria](https://track.editorialmanager.com/CL0/https:%2F%2Fjournals.plos.org%2Fglobalpublichealth%2Fs%2Fcriteria-for-publication/1/010f019679fe25f0-d017ae91-b392-4ae5-bacc-a5331a92c95d-000000/7Ucdr9-kDcBRQqMgpA7mkWW1DvIIkJ-V5l_ku38Drag=208)? Is the manuscript technically sound, and do the data support the conclusions? The manuscript must describe methodologically and ethically rigorous research with conclusions that are appropriately drawn based on the data presented.

Reviewer #1: Yes

2. Has the statistical analysis been performed appropriately and rigorously?

Reviewer #1: Yes

3. Have the authors made all data underlying the findings in their manuscript fully available (please refer to the Data Availability Statement at the start of the manuscript PDF file)?

The [PLOS Data policy](https://journals.plos.org/globalpublichealth/s/data-availability) requires authors to make all data underlying the findings described in their manuscript fully available without restriction, with rare exception. The data should be provided as part of the manuscript or its supporting information, or deposited to a public repository. For example, in addition to summary statistics, the data points behind means, medians and variance measures should be available. If there are restrictions on publicly sharing data—e.g. participant privacy or use of data from a third party—those must be specified.

Reviewer #1: No

Thank you for the comment. We have prepared the dataset in a suitable format (Excel file) and attached it as a supplementary file to accompany the published article on the journal website. This file includes relevant data underlying the findings reported in our manuscript.

4. Is the manuscript presented in an intelligible fashion and written in standard English?

PLOS Global Public Health does not copyedit accepted manuscripts, so the language in submitted articles must be clear, correct, and unambiguous. Any typographical or grammatical errors should be corrected at revision, so please note any specific errors here.

Reviewer #1: Yes

5. Review Comments to the Author

Please use the space provided to explain your answers to the questions above. You may also include additional comments for the author, including concerns about dual publication, research ethics, or publication ethics. (Please upload your review as an attachment if it exceeds 20,000 characters)

Reviewer #1: This is a well-planned analysis looking at the association between Charlson Comorbidity Index and positive blood cultures. Detailed and well-written.

What is the rationale for choosing 2 days after admission as a cut-off for hospital acquired bacteremia? Is 2 days a long enough duration for someone to acquire a hospital infection?

Thank you for this important question. The threshold of two calendar day after hospital admission is a widely accepted criterion used to differentiate community-acquired from hospital-acquired infections. This cut-off is consistent with the World Health Organization’s GLASS (Global Antimicrobial Resistance Surveillance System) protocol, which defines hospital-acquired infections as those occurring after two calendar days of hospital admission, under the assumption that the infection was not incubating at the time of admission.

This time frame is based on the typical incubation period of many nosocomial pathogens and the understanding that infections identified from blood specimens taken within the first two calendar days of hospital admission are more likely to be community-acquired. While some hospital-acquired infections may develop after a shorter duration, the ≥2-calendar day definition offers a standardized and practical approach for surveillance and has been widely used in global and national reporting systems, including in LMICs.

Hospital-acquired infection is briefly mentioned in the abstract and introduction, but not really expanded in the discussion. Needs to be include to tie the manuscript together. How the use of CCI for hospital acquired infection different from its application in patient with community acquired infections who get hospitalized?

Thank you for your suggestion. The application of the Charlson Comorbidity Index (CCI) may differ between patients with hospital-acquired infections (HAIs) and those hospitalized with community-acquired infections (CAIs). In patients with HAIs, CCI may be less predictive of infection risk because hospital-related factors—such as exposure to invasive procedures, ICU admission, length of stay, and exposure to antimicrobial-resistant pathogens—play a dominant role in infection acquisition. Thus, its utility in predicting positive blood culture results for HAIs may be limited in settings where nosocomial risks and healthcare-associated exposures are the primary drivers of infection. In contrast, in patients with CAIs, the comorbidity burden in these cases may influence the patient’s immune response and initial risk of infection upon presentation.

In our study, we didn’t included CAIs because as a tertiary referral hospital, almost patients in our setting were transferred from other facilities, often after receiving prior antibiotic therapy. As a result, it was difficult to identify patients with true community-acquired infections. Further research is needed to explore the relationship between CCI and patients with CAIs. We have added this point to the discussion section.

Abstract
- Is Low- and Middle-Income Countries, Intensive/High Care Unit all capital letters? – And throughout the rest of the manuscript, revise what needs to be capitalized.

Thank you. We have revised the manuscript to ensure correct capitalization throughout, including changing “Low- and Middle-Income Countries” and “Intensive/High Care Unit” to lowercase, as appropriate.

- Line 38 – 40 include % if word count allows

Thank you. We have included the percentages in Line 38–40 as suggested.

- Line 43 – 45, the OR would be more informative than p-value alone.

Thank you for the valuable suggestion. We agree that including the odds ratios would enhance the interpretability of our findings. However, due to the journal's strict word count limit for the abstract (maximum 300 words), we are unable to add both odds ratios and p-values without exceeding the allowed length. To address this, we have prioritized reporting p-values in the abstract and have included the corresponding odds ratios in the main results section of the manuscript.

- Improve conclusion of abstract – what other factors? Are these conclusions you can directly make from your results?
Thank you. We have revised the conclusion.

Introduction
- Line 52-53, reference?

Thank you. We have added the appropriate reference to support this statement.

- Once you have introduced the acronym, no need to include the full form? - line 71 and through methods and other sections for other acronyms as well.

Thank you. We have revised the manuscript to use acronyms consistently after their first mention, including in the Methods and other sections.

Methods:
- Well described analysis methods.
- Although the methods are referenced, short description of this hierarchy rule would be helpful to understand scoring.

Thank you for the suggestion. We have added a brief description of the hierarchy rule in the Methods section.

- Can you comment on the completeness and quality of the electronic records?
Thank you for the comment. We have addressed the completeness and quality of the electronic records in the result and discussion section, noting that the amount of missing data was minimal and unlikely to affect the overall findings.

- What are progressive wards?

Progressive wards (also known as progressive care units or step-down units) are hospital units that provide intermediate care for patients who no longer need intensive care (ICU) but still require more monitoring and support than provided in general wards.

- What is the minimum and maximum CCI value you can get, rationale for the categorization?

In our dataset, observed CCI scores ranged from 0 to 11. For analysis, we categorized CCI into three groups (0, 1, and >1) to reflect increasing comorbidity burden while maintaining sufficient sample size in each category. This categorization also provided a better model fit based on the likelihood ratio test (LRT).

Results
- Patient-days calculation not described in the methods – please include.

Thank you. We have added a brief description of patient days and blood culture utilization rate in the methods section.

- Add percent for 3875 – line 171

Thank you for the suggestion. We have added the corresponding percentage for 3,875 in line 171.

- What was the median (range) blood cultures per participant?

The median number of blood cultures collected per patient after 48hours of admission was 1 (IQR: 1–1; range: 1–7)

- Line 178, add %

Thank you. We have added the %.

- Line 179 – 181 should be in the methods

Thank you for the suggestion. We have moved the text in Lines 179–181 to the Methods section as recommended.

- Line 190-191, 371/786=47.2%?

Thank you for pointing this out. We have double-checked the calculation and corrected the percentage to 47.2%

- Line 191, 192, add %

Thank you. We have included the percentages in Line 191–192 as suggested.

- Describe figures 3 and 4 any further? Figures need to be clear stand alone, add information as appropriate

Thank you for the suggestion. We have revised the legend for Figure 3 and 4 to include additional details on the data presented

- Line 208 – 214, what proportion of participants have more than one comorbidity?
Of the 3,875 patients, 1,591 (41.1%) had no comorbidities, 1,738 (44.9%) had one comorbidity, 448 (11.6%) had two comorbidities, and 98 (2.5%) had more than two comorbidities.

- Minor comment, could make the tables cleaner by not having % in each row/col in the results.

Thank you for the suggestion. We had revised table 1 as suggested.

Discussion:
- Line 288-294 not described in results. – clarify the paragraph. Not clear what the author means. How does it relate to results of the analysis?

Thank you. We refer to the AMR data from our previous research which used the same data source, but the sample selection for analysis differed. Therefore, we do not include the results in the results section. Prior antibiotic use may reduce blood culture positivity, particularly when pathogens are susceptible to the administered agents. However, frequent or inappropriate antibiotic exposure can promote the emergence and selection of antimicrobial-resistant organisms, contributing to the high burden of AMR. We have revised the paragraph.

- Line 295-297 Is not clear, does the author mean the CCI is not a risk factor for CLASBSI or was it the independent variable being studied?

The author means that the “total CCI” (sum of all points for each comorbidity) was not strongly associated with increased risk for CLABSI. However, the individual patient's comorbid conditions were strongly associated with either increased or decreased risk for CLABSI

6. PLOS authors have the option to publish the peer review history of their article ([what does this mean?](https://track.editorialmanager.com/CL0/https:%2F%2Fjournals.plos.org%2Fglobalpublichealth%2Fs%2Feditorial-and-peer-review-process%23loc-peer-review-history/1/010f019679fe25f0-d017ae91-b392-4ae5-bacc-a5331a92c95d-000000/l2jq9w80IdUKpAZsl-aHqRcj3WCEVEg3mUCL4No_W6g=208)). If published, this will include your full peer review and any attached files.


**Do you want your identity to be public for this peer review?** If you choose “no”, your identity will remain anonymous but your review may still be made public.


For information about this choice, including consent withdrawal, please see our [Privacy Policy](https://track.editorialmanager.com/CL0/https:%2F%2Fwww.plos.org%2Fprivacy-policy/1/010f019679fe25f0-d017ae91-b392-4ae5-bacc-a5331a92c95d-000000/D3G3qzgEPu9-Bvu17TSkmDNjVLDFYQWUoAQlTLM7XeI=208).

Reviewer #1: No

I consent to making my identity public for this peer review and agree to the publication of the peer review history alongside the article.

[NOTE: If reviewer comments were submitted as an attachment file, they will be attached to this email and accessible via the submission site. Please log into your account, locate the manuscript record, and check for the action link "View Attachments". If this link does not appear, there are no attachment files.]

While revising your submission, please upload your figure files to the Preflight Analysis and Conversion Engine (PACE) digital diagnostic tool, https://pacev2.apexcovantage.com/. PACE helps ensure that figures meet PLOS requirements. To use PACE, you must first register as a user. Registration is free. Then, login and navigate to the UPLOAD tab, where you will find detailed instructions on how to use the tool. If you encounter any issues or have any questions when using PACE, please email PLOS at figures@plos.org. Please note that Supporting Information files do not need this step.
